# Contact Electrification by Quantum-Mechanical Tunneling

**DOI:** 10.34133/2019/6528689

**Published:** 2019-08-04

**Authors:** Morten Willatzen, Zhong Lin Wang

**Affiliations:** ^1^CAS Center for Excellence in Nanoscience, Beijing Key Laboratory of Micro-Nano Energy and Sensor, Beijing Institute of Nanoenergy and Nanosystems, Chinese Academy of Sciences, Beijing 100083, China; ^2^School of Nanoscience and Technology, University of Chinese Academy of Sciences, Beijing 100049, China; ^3^School of Materials Science and Engineering, Georgia Institute of Technology, Atlanta, GA 30332-0245, USA

## Abstract

A simple model of charge transfer by loss-less quantum-mechanical tunneling between two solids is proposed. The model is applicable to electron transport and contact electrification between e.g. a metal and a dielectric solid. Based on a one-dimensional effective-mass Hamiltonian, the tunneling transmission coefficient of electrons through a barrier from one solid to another solid is calculated analytically. The transport rate (current) of electrons is found using the Tsu-Esaki equation and accounting for different Fermi functions of the two solids. We show that the tunneling dynamics is very sensitive to the vacuum potential versus the two solids conduction-band edges and the thickness of the vacuum gap. The relevant time constants for tunneling and contact electrification, relevant for triboelectricity, can vary over several orders of magnitude when the vacuum gap changes by one order of magnitude, say, 1 Å to 10 Å. Coulomb repulsion between electrons on the left and right material surfaces is accounted for in the tunneling dynamics.

## 1. Introduction

Contact electrification has been known since ancient times but the underlying fundamental mechanism is still not known [1–10]. The role of electron transport versus ion transport for the charge transfer has been under dispute [3, 7, 11–14], and only recently did work in the group of Zhong Lin Wang [15] assert that electron transport is the dominant triboelectricity mechanism for metal-metal, metal-semiconductor, and metal-insulator systems in contact.

Triboelectric charging is important to understand and control. On the one hand, it may have severe negative implications and is known to lead to explosion/fire [16], damage microelectronic components [17, 18], disturb fluid flow [19], and be able to increased friction and energy dissipation [20, 21] etc. On the other hand, since energy associated with low-frequency wave motion is abundant in nature as well as due to human activity, there is significant benefit to society in harvesting the energy in an effective way. Triboelectric nanogenerators (TENG's) are expected to be the primary candidates for energy generation since they can be easily implemented in a vast number of electronic devices. TENGs out-compete electromagnetic generators as the latter are ineffective at harvesting low-frequency (<5 Hz) [22–26] wave energy from, e.g. ocean, wind, and human motion.

While a classic macroscopic understanding of energy generation due to relative motion of systems of dielectric materials, through the concept of Maxwell's electric displacement, exists to a certain extent, the physical mechanisms and properties that play a role for the electron transfer processes in nanosystems are much less explored. Electron transfer involving photons (photoelectron emission) in nanoscale contact electrification has been demonstrated experimentally [14, 27–31]. The authors recently presented a mechanism for contact electrification and photon emission in atomic systems [32] based on Einstein's two-level system rate equations [33, 34], and in solid systems [35] using standard theory of electronic structure and optical properties of solids [36–38]. In the present work, we describe a simple loss-less electron transfer mechanism based on the Tsu-Esaki tunneling theory [39, 40] between dissimilar solids and show that the tunneling rate and contact electrification are strongly sensitive to the conduction-band edges of the two materials, the vacuum potential and the vacuum-gap thickness, and the Fermi levels. For simplicity, non-ideal processes such as charge escape from surfaces due to electric conduction of non-vacuum environment, or inside triboelectric materials, which are nonideal insulators, are neglected.

## 2. Carrier Concentrations and Fermi Levels

**Figure 1:**
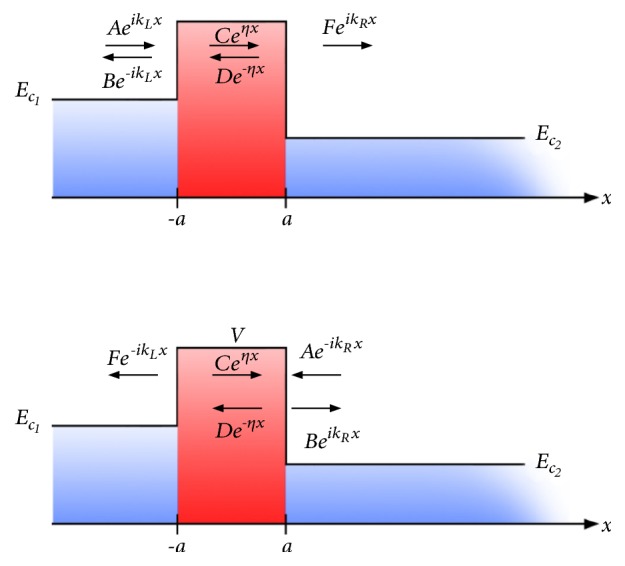
Band diagram for tunneling of electrons through a vacuum gap in the absence of a Coulomb repulsion potential. (a) Left-to-right tunneling and (b) right-to-left tunneling.

Consider a structure (Figure 1) consisting of two materials denoted by subscripts 1 (left) and 2 (right) with conduction-band edges *E*_*c*1_ and *E*_*c*2_, respectively. A vacuum gap of thickness 2*a* exists between them and acts as a barrier (of potential *V*) for electron transfer. For simplicity, we shall assume an effective mass approximation for electrons and consider the effective mass to be the same, *m*^*∗*^, everywhere in the structure; i.e.,

(1) $$\begin{array}{c} -\frac{\hbar^{2}}{2m^{*}}\frac{d^{2}\psi }{dx^{2}}+V_{pot}\left(\;x\;\right)\psi \left(\;x\;\right)=E\psi \left(\;x\;\right), \end{array}$$

where *E* is the electron energy and

(2) Vpotx=Ec1,x<−a,Vpotx=V,−a<x<a,Vpotx=Ec2,x>a.

For a general form of the potential *V*_*pot*_(*x*), a simple and frequently applied approximation to obtain the tunneling coefficient is to solve the Schrödinger equation (1) using the Wentzel-Kramers-Brillouin (WKB) approximation [41–43]. In the important case of a linear barrier potential, Gundlach [44] derived a simple expression for the tunneling coefficient. In the following, we present a matrix scheme that can be extended rather easily to any barrier potential function *V*_*pot*_(*x*). We give the full details for the most important cases of a flat and a linear barrier potential.

## 3. Tunneling and Coulomb Effects

The electron number *N*_1_ in the left material is

(3) $$\begin{array}{c} N_{1}=\left(\;2\;\right)\underset{k}{\sum }f_{1}\left(\;k\;\right)=\left(\;2\;\right)\int \frac{dk}{8\pi^{3}/V}f_{1}\left(\;k\;\right), \end{array}$$

where prefactor (2) is due to the spin degeneracy, so the number of electrons per area is

(4) $$\begin{array}{c} \frac{N_{1}}{A}=\frac{L_{x}}{4\pi^{3}}\int dkf_{1}\left(\;k\;\right)=\frac{L_{x}}{\pi^{2}}\overset{\infty }{\underset{0}{\int }}k^{2}f_{1}\left(\;k\;\right)dk. \end{array}$$

Here, we have considered the interface to vacuum to be perpendicular to the *x* axis and denote the thickness of the left material *L*_*x*_. The left material's total volume is then *V* = *AL*_*x*_ and *f*_1_(*k*) is the Fermi function

(5) $$\begin{array}{c} f_{1}\left(\;k\;\right)=\frac{1}{1+e^{\left(E_{c1}-e\phi_{L}+\hbar^{2}k^{2}/2m^{*}-E_{F1}\right)/\left(k_{B}T\right)}}, \end{array}$$

where *E*_*F*1_ is the Fermi energy of the left material, −*eϕ*_*L*_ is the electron energy contribution in the presence of an electric potential *ϕ*_*L*_ in the left material, *T* is the absolute temperature, and *k*_*B*_ is the Boltzmann constant. Further, parabolic dispersion of the conduction electrons is assumed:

(6) $$\begin{array}{c} E_{L}=E_{c1}-e\phi_{L}+\frac{\hbar^{2}k^{2}}{2m^{*}}. \end{array}$$

This allows us to rewrite the electron number per area in the left material as

(7) N1A=Lx2π22m∗ħ23/2∫Ec1−eϕL∞E−Ec1−eϕLf1EdE,

where

(8) $$\begin{array}{c} f_{1}\left(\;E\;\right)=\frac{1}{1+e^{\left(E-E_{F1}\right)/\left(k_{B}T\right)}}. \end{array}$$

Similarly, we can write the electron number per area in the right material as

(9) N2ALx2π22m∗ħ23/2·∫Ec2−eϕR∞E−Ec2−eϕRf2EdE,

where −*eϕ*_*R*_ is the electron energy contribution in the presence of an electric potential *ϕ*_*R*_ in the right material, *E*_*F*2_ is the Fermi energy of the right material

(10) $$\begin{array}{c} f_{2}\left(\;E\;\right)=\frac{1}{1+e^{\left(E-E_{F2}\right)/\left(k_{B}T\right)}}, \end{array}$$

and the dispersion is

(11) $$\begin{array}{c} E_{R}=E_{c2}-e\phi_{R}+\frac{\hbar^{2}k^{2}}{2m^{*}}. \end{array}$$

Here, it is tacitly assumed the thickness (*L*_*x*_), effective mass, and temperature to be the same in the two materials. Note that the electric potentials *ϕ*_*L*_ and *ϕ*_*R*_ are different due to charge separation across the vaccum gap (will be shown in the following). Further, we shall assume that the electric potential is constant *ϕ*_*L*_ (*ϕ*_*R*_) in the left (right) material.

### 3.1. Symmetry of the Transmission Coefficient

Consider the general case with plane waves propagating to the left and right of the barrier

(12) ψx=AeikLx+Be−ikLx,x<−a,ψx=FeikRx+Ge−ikRx,x>a.

The probability current *J* is defined as

(13) $$\begin{array}{c} J=\frac{\hbar }{2m^{*}i}\left(\;\psi^{*}\frac{\partial \psi }{\partial x}-\psi \frac{\partial \psi^{*}}{\partial x}\;\right), \end{array}$$

thus the current density to the left of the barrier is

(14) $$\begin{array}{c} J_{L}=\frac{\hbar k_{L}}{m^{*}}\left(\;{\left|\;A\;\right|}^{2}-{\left|\;B\;\right|}^{2}\;\right), \end{array}$$

while the current density to the right of the barrier is

(15) $$\begin{array}{c} J_{R}=\frac{\hbar k_{R}}{m^{*}}\left(\;{\left|\;F\;\right|}^{2}-{\left|\;G\;\right|}^{2}\;\right). \end{array}$$

Particle conservation implies that the number of particles entering the material to the left of the barrier is equal to the number of particles leaving the material to the right of the barrier; i.e.,

(16) $$\begin{array}{c} J_{L}=J_{R}, \end{array}$$

so

(17) $$\begin{array}{c} k_{L}\left(\;{\left|\;A\;\right|}^{2}-{\left|\;B\;\right|}^{2}\;\right)=k_{R}\left(\;{\left|\;F\;\right|}^{2}-{\left|\;G\;\right|}^{2}\;\right), \end{array}$$

or

(18) $$\begin{array}{c} k_{L}{\left|\;B\;\right|}^{2}+k_{R}{\left|\;F\;\right|}^{2}=k_{L}{\left|\;A\;\right|}^{2}+k_{R}{\left|\;G\;\right|}^{2}. \end{array}$$

The latter expression can be written is matrix form as

(19) $$\begin{array}{c} \left[\begin{array}{c} \sqrt{k_{L}}B\\ \sqrt{k_{R}}F \end{array}\right]=\left[\begin{array}{cc} S_{11} & S_{12}\\ S_{21} & S_{22} \end{array}\right]\left[\begin{array}{c} \sqrt{k_{L}}A\\ \sqrt{k_{R}}G \end{array}\right], \end{array}$$

where the 2 × 2 matrix *S* couples outgoing waves to incoming waves (scattering matrix form). Defining

(20) $$\begin{array}{c} \Psi^{in}=\left[\begin{array}{c} \sqrt{k_{L}}A\\ \sqrt{k_{R}}G \end{array}\right], \end{array}$$

and

(21) $$\begin{array}{c} \Psi^{out}=\left[\begin{array}{c} \sqrt{k_{L}}B\\ \sqrt{k_{R}}F \end{array}\right], \end{array}$$

Equation (18) implies

(22) $$\begin{array}{c} {\Psi^{out}}^{†}\Psi^{out}={\Psi^{in}}^{†}\Psi^{in}; \end{array}$$

i.e.,

(23) $$\begin{array}{c} {\Psi^{in}}^{†}S^{†}S\Psi^{in}={\Psi^{in}}^{†}\Psi^{in}, \end{array}$$

so

(24) $$\begin{array}{c} S^{†}S=I. \end{array}$$

Since the barrier potential is real, the system possess time-reversal symmetry, i.e., *ψ*^*∗*^(*x*) is a solution if *ψ*(*x*) is a solution. The time-reversed solution of Eq. (12) is

(25) ψ∗x=A∗e−ikLx+B∗eikLx,x<−a,ψ∗x=F∗e−ikRx+G∗eikRx,x>a.

The left and right solutions are related by the same *S* matrix, i.e.,

(26) $$\begin{array}{c} \left[\begin{array}{c} \sqrt{k_{L}}A^{*}\\ \sqrt{k_{R}}G^{*} \end{array}\right]=\left[\begin{array}{cc} S_{11} & S_{12}\\ S_{21} & S_{22} \end{array}\right]\left[\begin{array}{c} \sqrt{k_{L}}B^{*}\\ \sqrt{k_{R}}F^{*} \end{array}\right], \end{array}$$

and

(27) $$\begin{array}{c} {\Psi^{in}}^{*}=S{\Psi^{out}}^{*}, \end{array}$$

Thus, the relations

(28) Ψin∗=SΨout∗,Ψout=SΨin,

together yield the condition

(29) $$\begin{array}{c} S^{*}S=I. \end{array}$$

Hence, combining with (24),

(30) $$\begin{array}{c} S^{T}=S, \end{array}$$

and

(31) $$\begin{array}{c} T_{LR}={\left.\;\frac{k_{R}{\left|F\right|}^{2}}{k_{L}{\left|A\right|}^{2}}\;\right|}_{G=0}=S_{21}=S_{12}={\left.\;\frac{k_{L}{\left|B\right|}^{2}}{k_{R}{\left|G\right|}^{2}}\;\right|}_{A=0}=T_{RL}, \end{array}$$

and symmetry in the transmission coefficient is proved.

## 4. The Effect of Coulomb Repulsion

As electrons are transferred from the left to the right material, the surface charge on the left material piles up according to the dynamic expression

(32) $$\begin{array}{c} \sigma \left(\;t\;\right)=-e\frac{N_{1}\left(t\right)-N_{1}\left(t=0\right)}{A}, \end{array}$$

and the electric displacement obeys the Maxwell-Poisson equation

(33) $$\begin{array}{c} \frac{\partial D}{\partial x}=\sigma \left(\;t\;\right)\left(\;\delta \left(\;x+a\;\right)-\delta \left(\;x-a\;\right)\;\right), \end{array}$$

so

(34) D=0,if  x<−a,D=σt,if  −a<x<a,D=0,if  x>a.

In obtaining this we have tacitly assumed that charges are distributed on the surfaces in close proximity and not in the bulk part of the two materials. This assumption implies, strictly speaking, that the two materials are perfect conductors. If one or both materials are semiconductors or dielectrics, the transfered charge will likely be distributed near (but not necessarily on) the material surfaces and the electric displacement result above will be changed. We will, however, neglect this complication in the following.

Since *D* = *ϵE* = −*ϵ*(∂*ϕ*/∂*x*), where *ϕ* is the electric potential, we have in the vacuum region (*ϵ* = *ϵ*_0_),

(35) $$\begin{array}{c} \phi \left(\;x,t\;\right)=-\frac{\sigma \left(t\right)}{\epsilon_{0}}x-\frac{\sigma \left(t\right)}{\epsilon_{0}}a, \end{array}$$

setting *ϕ*(*x* = −*a*) = 0. This electric potential is repulsive as it opposes electron transfer between the two materials. The electron energy contribution *V*^*σ*^ = −*eϕ*(*x*, *t*) in the presence of the Coulomb repulsion potential is therefore

(36) Vσx,t=0,if  x<−a,Vσx,t=eσtϵ0x+eσtϵ0a,if  −a<x<a,Vσx,t=2eσtϵ0a,if  x>a.

The effect of the Coulomb repulsion potential is that the tunneling barrier in the vacuum region shifts from a constant potential *V* to a linearly increasing barrier potential *V* + *V*^*σ*^(*x*, *t*) = *V* + *e*(*σ*(*t*)/*ϵ*_0_)*x* + *e*(*σ*(*t*)/*ϵ*_0_)*a* with position *x*. Hence, the electron transfer rate decreases as the surface charge density increases and the net transfer of electrons will stop before the Fermi levels of the two materials become equal assuming a constant temperature everywhere in the system. If the Coulomb repulsion potential is negligibly small, net transfer of electrons first stops when the Fermi levels become equal.

## 5. Tunneling Transmission Probability

Let us derive expressions for the tunneling transmission probabilities *T*_*LR*_(*E*) in the absence and presence of Coulomb repulsion.

### 5.1. Tunneling Transmission Probability - Absence of a Coulomb Repulsion Potential

Let us derive expressions for the tunneling transmission probability *T*_*LR*_(*E*) in the absence of Coulomb repulsion. Firstly, with reference to Figure 1 and (1), the electron envelope function *ψ* is

(37) ψx=AeikLx+Be−ikLx,x<−a,ψx=Ceηx+De−ηx,−a<x<a,ψx=FeikRx,x>a,

where

(38) kL=2m∗E−Ec1ħ2,

(39) η=2m∗V−Eħ2,

(40) kR=2m∗E−Ec2ħ2.

Continuity of *ψ* and *dψ*/*dx* at *x* = −*a* and *x* = *a* yield

(41) Ae−ikLa+BeikLa=Ce−ηa+Deηa,

(42) ikLAe−ikLa−ikLBeikLa=ηCe−ηa−ηDeηa,

(43) Ceηa+De−ηa=Fe−ikRa,

(44) Cηeηa−Dηe−ηa=ikRFe−ikRa.

The transmission probability *T*_*LR*_ is

(45) $$\begin{array}{c} T_{LR}=\frac{v_{R}}{v_{L}}\frac{{\left|F\right|}^{2}}{{\left|A\right|}^{2}}=\frac{k_{R}}{k_{L}}\frac{{\left|F\right|}^{2}}{{\left|A\right|}^{2}}=k_{L}k_{R}{\left|\;\frac{4i\eta }{\left(-ik_{L}-\eta \right)\left(-ik_{R}-\eta \right)e^{-2\eta a}e^{i\left(k_{L}+k_{R}\right)a}-\left(-ik_{L}+\eta \right)\left(-ik_{R}+\eta \right)e^{2\eta a}e^{i\left(k_{L}+k_{R}\right)a}}\;\right|}^{2}, \end{array}$$

where *v*_*L*_ = *ħk*_*L*_/*m*^*∗*^ and *v*_*R*_ = *ħk*_*R*_/*m*^*∗*^.

### 5.2. Tunneling Transmission Probability - Presence of a Coulomb Repulsion Potential

The Schrödinger equation in the vacuum region accounting for Coulomb repulsion has the form

(46) $$\begin{array}{c} -\frac{\hbar^{2}}{2m^{*}}\frac{d^{2}\psi }{dx^{2}}+\left(\;V^{vac}+Fx\;\right)\psi \left(\;x\;\right)=E\psi \left(\;x\;\right), \end{array}$$

where, with reference to Eq. (36),

(47) F=eσϵ0,Vvac=V+eσϵ0a.

We note that here, and in the following, the surface charge density is a time-dependent function.

This equation can be recast as

(48) $$\begin{array}{c} \frac{d^{2}\psi }{dx^{2}}-\left(\;-\tilde{E}+{\tilde{V}}^{vac}+\tilde{F}x\;\right)\psi =0, \end{array}$$

where

(49) V~vac=2m∗ħ2Vvac,F~=2m∗ħ2F,E~=2m∗ħ2E.

Setting

(50) $$\begin{array}{c} z=\left(\;-\tilde{E}+{\tilde{V}}^{vac}+\tilde{F}x\;\right){\tilde{F}}^{-2/3}, \end{array}$$

allows rewriting Eq. (48) as Airy's equation:

(51) $$\begin{array}{c} \frac{d^{2}\psi }{dz^{2}}-z\psi \left(\;z\;\right)=0, \end{array}$$

for which the solution is

**Figure 2:**
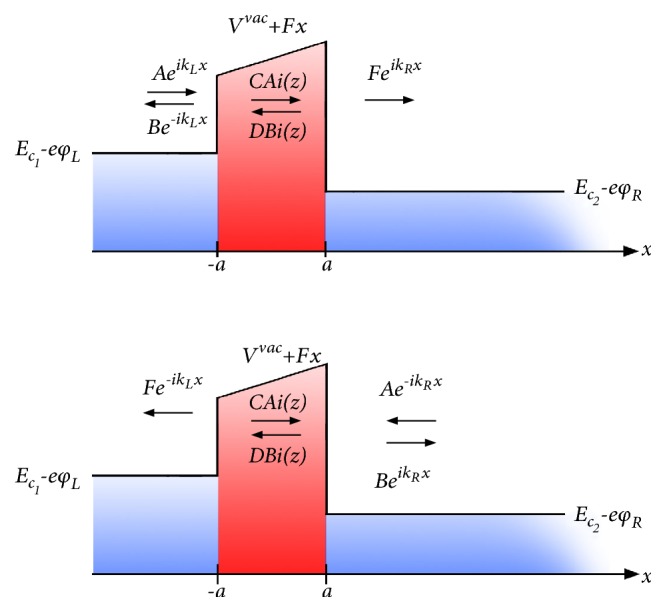
Band diagram for tunneling of electrons through a vacuum gap in the presence of a Coulomb repulsion potential. (a) Left-to-right tunneling and (b) right-to-left tunneling.

(52) ψzCAiz+DBiz=CAi−E~+V~vac+F~xF~−2/3+DBi−E~+V~vac+F~xF~−2/3,

where *Ai*(*Bi*) are Airy functions, and *C* and *D* are constants. Hence, with reference to Figure 2, the electron envelope function *ψ* in the whole domain can be written as

(53) ψx=AeikLx+Be−ikLx,x<−a,ψx=CAi−E~+V~vac+F~xF~−2/3+DBi−E~+V~vac+F~xF~−2/3,−a<x<a,ψx=FeikRx,x>a,

where

(54) kL=2m∗E−Ec1−eϕLħ2,

(55) kR=2m∗E−Ec2−eϕRħ2.

Let us consider, as shown in Figure 2, that *E*_*c*1_ > *E*_*c*2_. Transport left-to-right and right-to-left can then only take place for energies *E* ≥ *E*_*c*1_ (*ϕ*_*L*_ = 0).

Continuity of *ψ* and *dψ*/*dx* at *x* = −*a* and *x* = *a* yields

(56) Ae−ikLa+BeikLa=CAi−E~+V~vac−F~aF~−2/3+DBi−E~+V~vac−F~aF~−2/3,ikLAe−ikLa−ikLBeikLa=CdAi−E~+V~vac+F~xF~−2/3dxx=−a+DdBi−E~+V~vac+F~xF~−2/3dxx=−a,Fe−ikRa=CAi−E~+V~vac+F~aF~−2/3+DBi−E~+V~vac+F~aF~−2/3,ikRFe−ikRa=CdAi−E~+V~vac+F~xF~−2/3dxx=a+DdBi−E~+V~vac+F~xF~−2/3dxx=a.

The transmission probability *T*_*LR*_ is again

(57) $$\begin{array}{c} T_{LR}=\frac{v_{R}}{v_{L}}\frac{{\left|F\right|}^{2}}{{\left|A\right|}^{2}}=\frac{k_{R}}{k_{L}}\frac{{\left|F\right|}^{2}}{{\left|A\right|}^{2}}, \end{array}$$

where *v*_*L*_ = *ħk*_*L*_/*m*^*∗*^, *v*_*R*_ = *ħk*_*R*_/*m*^*∗*^. By setting *A* = 1 and solving the 4 × 4 matrix Eq. (56) for *F*, the transmission probability *T*_*LR*_ can be determined.

For the reverse transmission probability, *T*_*RL*_ we already know that *T*_*RL*_(*E*) = *T*_*LR*_(*E*).

## 6. Tunneling Current

We can now write down an expression for the tunneling current. Consider electrons in the left material moving to the right. The velocity component perpendicular to the interface with vacuum is *v* = *ħk*_*x*,1_/*m*^*∗*^ so the electron (particle) current is

(58) IL=21Lx·∑kx,1>0ħkx,1m∗TLREkx,1∑k||f1E1−f2E,

where *T*_*LR*_(*E*(*k*_*x*,1_)) is the tunneling transmission coefficient assumed to only depend on the momentum perpendicular to the interface, 1/*L*_*x*_ is the electron density (plane wave normalized over *L*_*x*_), and *E* = *E*(*k*_*x*,1_). Here, we have used that current is given by charge times velocity times density.

There is also a flow from the right material to the left (of opposite sign):

(59) IR=21Lx·∑kx,2<0ħkx,2m∗TRLEkx,2∑k||f2E1−f1E,

where *E* = *E*(*k*_*x*,2_) so the net particle flow is

(60) $$\begin{array}{c} I=I_{L}+I_{R}. \end{array}$$

The transmission coefficients obey the condition

(61) $$\begin{array}{c} 1=T_{LR}+R_{LR}=T_{RL}+R_{RL}. \end{array}$$

Above we proved that the transmission coefficient is symmetric, i.e.,

(62) $$\begin{array}{c} T_{LR}\left(\;E\left(\;k_{x,1}\;\right)\;\right)=T_{RL}\left(\;E\left(\;k_{x,2}\;\right)\;\right)\equiv T\left(\;E\;\right). \end{array}$$

The particle current density per area can then be written as

(63) JIA=21ALx∑kx,1>0ħkx,1m∗TE∑k||f1E1−f2E+21ALx∑kx,2<0ħkx,2m∗TE∑k||f2E1−f1E.

Using

(64) ∑k|| =∫0∞2πk||dk||4π2/A,∑kx,1>0 =∫0∞dkx,12π/Lx,∑kx,2<0 =∫−∞0dkx,22π/Lx,

the particle current density per area can be written as

(65) J=IA=214π2ħm∗·∫0∞dkx,1kx,1TE∫0∞k||dk||f1E1−f2E−214π2ħm∗·∫0∞dkx,2kx,2TE∫0∞k||dk||f2E1−f1E.

Then, since

(66) $$\begin{array}{c} E=E_{x}+E_{||}, \end{array}$$

where

(67) Ex=Ec1−eϕL+ħ2kx,122m∗=Ec2−eϕR+ħ2kx,222m∗,E||=ħ2k||22m∗,

it follows that

(68) dEx=ħ2m∗kx,1dkx,1=ħ2m∗kx,2dkx,2,dE||=ħ2m∗k||dk||.

and Eq. (66) can be written as

(69) J22πm∗h3·∫EminVdExTEx∫0∞dE||f1E−f2E,

where we introduced *E*_*min*_ as the lower *E*_*x*_ integration limit to reflect that tunneling is only possible above the energy of the highest conduction band edge of the two materials. Similarly, as upper *E*_*x*_ integration limit, the vacuum barrier edge *V* is introduced.

This expression can be further simplified by use of

the integral expression

(70) $$\begin{array}{c} \int \frac{dx}{1+exp\left(x\right)}=ln\left(\;\frac{1}{1+exp\left(-x\right)}\;\right)+C, \end{array}$$

to get

(71) $$\begin{array}{c} J=\left(\;2\;\right)2\pi \frac{m^{*}}{h^{3}}\overset{V}{\underset{E_{min}}{\int }}dE_{x}T\left(\;E_{x}\;\right)D\left(\;E_{x}\;\right), \end{array}$$

where

(72) $$\begin{array}{c} D\left(\;E_{x}\;\right)=k_{B}T\ln\left(\;\frac{1+\exp\left(-\left(E_{x}-E_{F1}\right)/k_{B}T\right)}{1+\exp\left(-\left(E_{x}-E_{F2}\right)/k_{B}T\right)}\;\right). \end{array}$$

## 7. Equation Framework

We are now in a position to formulate a mathematical modeling framework for the transfer of electrons between two distinct solids separated by a vacuum gap. The charge transfer between the two solids is described by the following equations

(73) dN1/Adt=−J,

(74) dN2/Adt=J,

where *N*_1_/*A*, *N*_2_/*A*, and *J* are determined by use of Equations (4), (9), (57), and (71)-(72). Obviously, electron conservation in the whole structure is automatically guaranteed.

### 7.1. Numerical Scheme

Given initial values *E*_*F*1_^0^ and *E*_*F*2_^0^ at time *t* = *t*^0^, expressions for *N*_1_^0^, *N*_2_^0^, *J*^0^ are determined. At time

(75) $$\begin{array}{c} t^{i+1}=t^{i}+\Delta t,\;i=0,1,2,3,\ldots , \end{array}$$

where Δ*t* is chosen sufficiently small to guarantee accuracy and stability in the numerical scheme, new values *N*_1_^*i*+1^, *N*_2_^*i*+1^ are found from

(76) N1i+1=N1i+AΔt·−Ji,

(77) N2i+1=N2i+AΔt·Ji,

from which updated values *E*_*F*1_^*i*+1^ = *E*_*F*1_^*i*+1^(*N*_1_^*i*+1^) and *E*_*F*2_^*i*+1^ = *E*_*F*2_^*i*+1^(*N*_2_^*i*+1^) are determined. The new electron tunneling current *J*^*i*+1^ is then given by Equations (71)-(72). Further:

(78) N1i+2=N1i+1+AΔt−Ji+1,

(79) N2i+2=N2i+1+AΔtJi+1,

etc. This concludes the equation framework to determine the temporal evolution of electron tunneling transfer through a vacuum gap.

## 8. Numerical Test Cases for Electron Transfer by Tunneling

In this section, we will calculate electron transfer dynamics by tunneling through a barrier defined by a vacuum gap. The tunneling efficiency depends strongly on the value of the vacuum potential vs. the conduction band edges of the left and right solids. It also depends strongly on the thickness of the vacuum gap.

### 8.1. Case 1: Varying the Thickness of the Vacuum Gap and Fixing the Vacuum Potential to 2 eV

We consider the following parameter values: *m*^*∗*^ = 0.1*m*_0_ where *m*_0_ is the free-electron mass, *L* = 100 Å, *E*_*c*1_ = 0 eV, *E*_*c*2_ = −0.4 eV, *V* = 2 eV, the inital Fermi levels *E*_*F*1_ = *E*_*c*1_ + 0.01 eV, *E*_*F*2_ = *E*_*c*1_ − 0.1 eV, *T* = 300 K, and the vacuum gap thickness 2*a* is varied between 4 Å and 20 Å.

**Figure 3:**
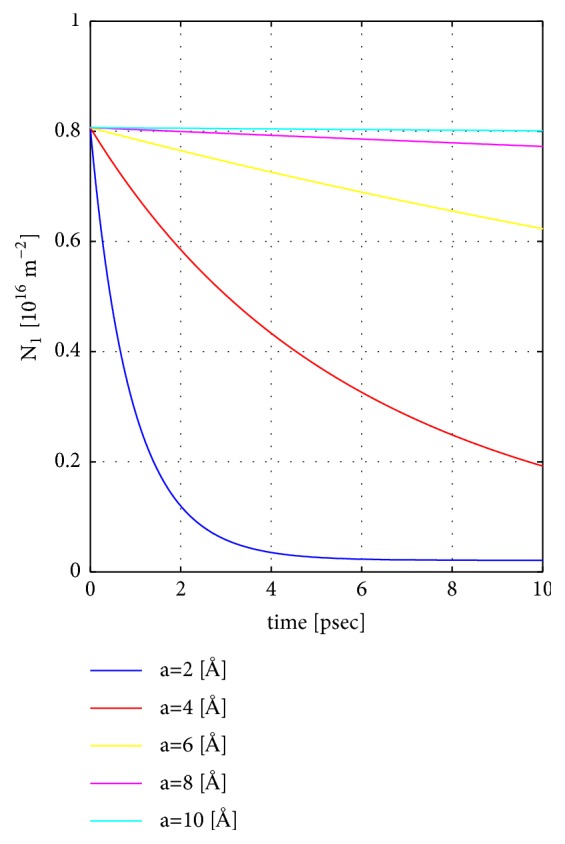
Case 1: Time dependence of the electron number in the left material *N*_1_ as a function of time *t* for a set of vacuum gap thicknesses 2*a*. The vacuum potential *V* is fixed to 2 eV. Other parameters are given in the main text.

In Figure 3, we show the temporal variation of the electron number (arbitrary unit) for a set of different vacuum-gap thicknesses. It's clear that the electron transfer drops strongly with the increase of the vacuum gap thickness. Observe the small time scale. Later, we will demonstrate electron transfer on a much slower time scale for the case of a larger vacuum potential. Note that *N*_1_ + *N*_2_ is a constant as a function of time, i.e., overall charge conservation is guaranteed. If the left and right solids initially are uncharged, the left solid becomes increasingly charged with time according to

(80) $$\begin{array}{c} Q\left(\;t\;\right)=e\left(\;N_{1}\left(\;t\;\right)-N_{1}\left(\;t=0\;\right)\;\right). \end{array}$$

Similarly the charge on the right solid is −*Q*(*t*) as a function of time *t*. The build-up of surface charges opposes the net transfer of electrons until the net electron transfer until a quasi-equilibrium situation is established whereafter the electron densities in the left and right materials are constants.

### 8.2. Case 2: Varying the Potential of the Vacuum Barrier and Fixing the Distance 2*a* between the Materials to 10 Å.

**Figure 4:**
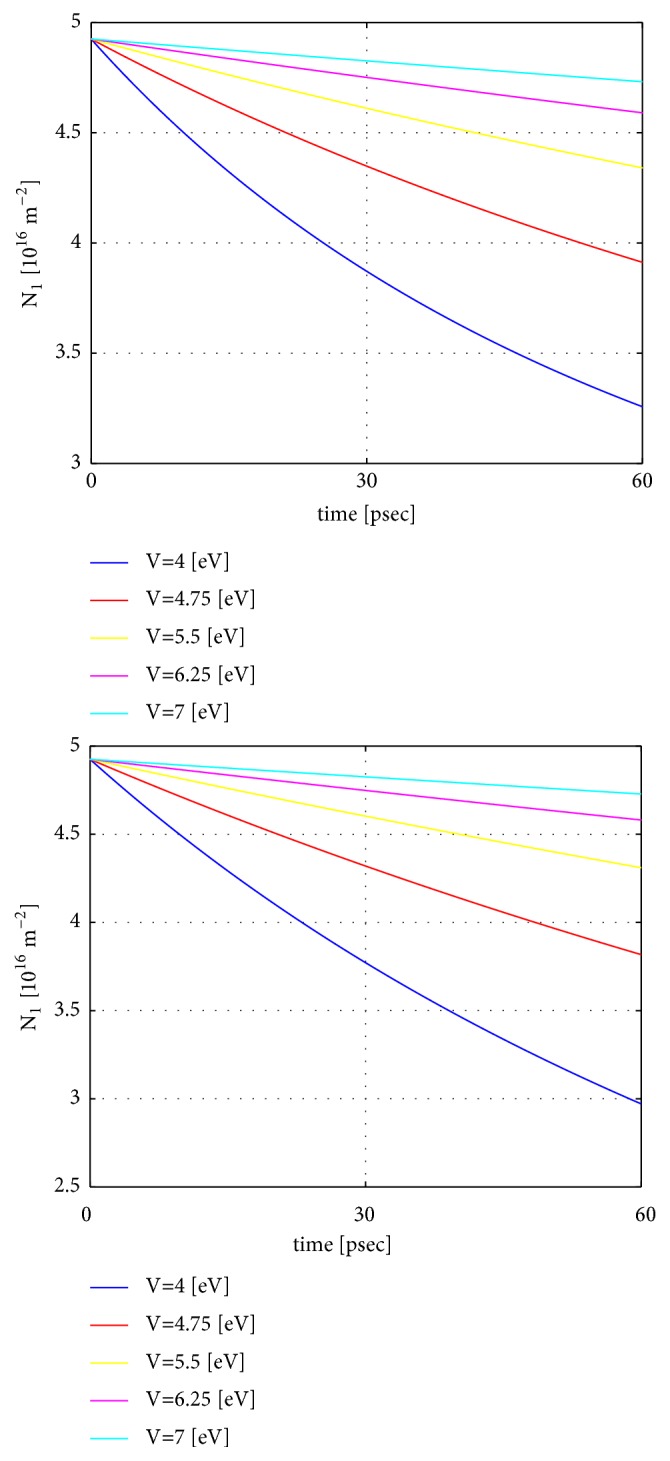
Case 2: Time dependence of the electron number in the left material *N*_1_ as a function of time *t* for a set of potential values *V*. The upper (lower) plot is with (without) the effect of Coulomb repulsion due to electron transfer and effective charging of the two materials. The distance between the materials 2*a* is fixed to 10 Å. Other parameters are given in the main text.

Next, we examine the effect of changing the vacuum potential *V* while fixing the vacuum-gap thickness 2*a* to 10 Å. The initial Fermi levels are *E*_*F*1_ = 0.1 eV and *E*_*F*2_ = −0.1 eV. The other parameters are the same as in case 1. Again, we see (Figure 4), that the electron transfer rate drops by several orders of magnitude as the vacuum potential is increased from 3 eV to 7 eV. The initial carrier density is approximately 4.9 · 10^16^  m^−2^ for all vacuum potential values. The upper (lower) plots correspond to cases with and without the influence of Coulomb repulsion, respectively. The effect of Coulomb repulsion is most pronounced for the smallest potential barrier *V* = 3 eV. Evidently, the carrier density *N*_1_ is higher in the case with Coulomb repulsion as compared to the case without Coulomb repulsion and most noticeably at times above approximately 30 psec.

### 8.3. Case 3: Varying the Potential of the Vacuum Barrier and Fixing the Distance 2*a* between the Materials to 20 Å.

**Figure 5:**
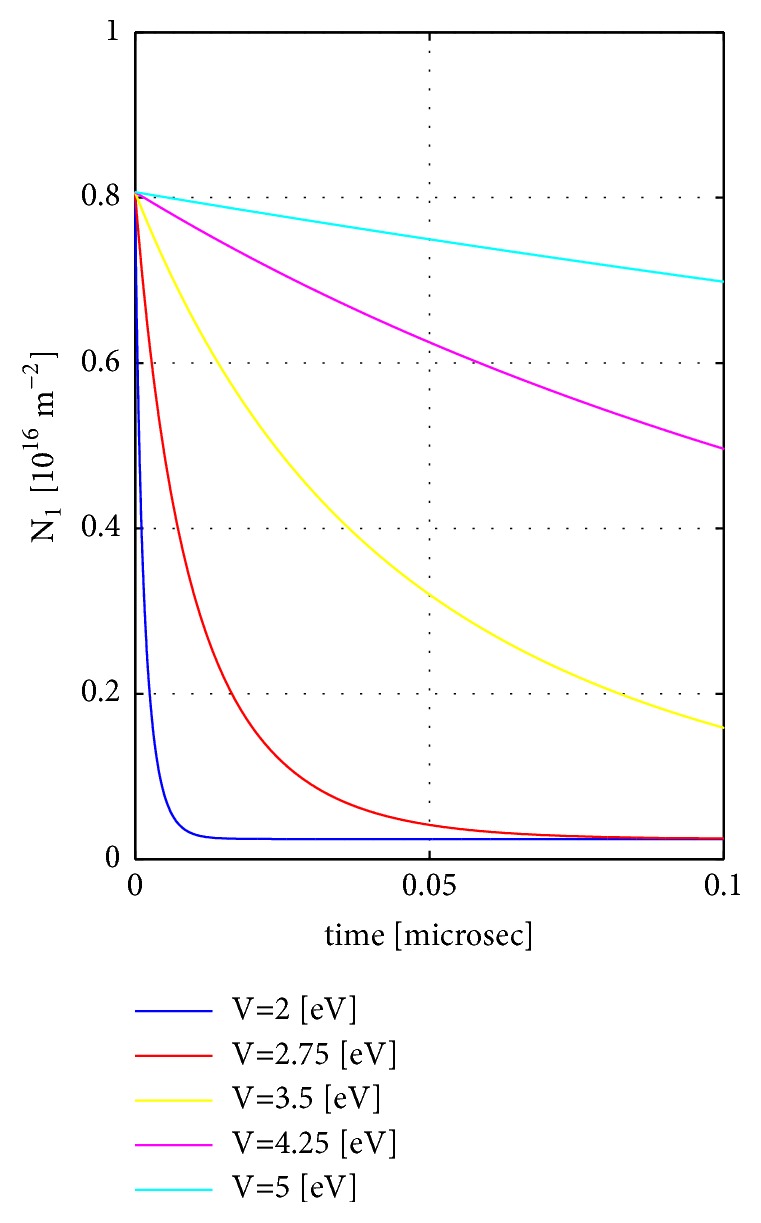
Case 3: Time dependence of the electron number in the left material *N*_1_ as a function of time *t* for a set of vacuum potential values *V*. The distance between the materials 2*a* is fixed to 20 Å. Other parameters are given in the main text.

We then examine the effect of changing the vacuum potential *V* between 2 eV and 5 eV while fixing the vacuum-gap thickness 2*a* to 20 Å. The initial Fermi levels are again *E*_*F*1_ = 0.01 eV and *E*_*F*2_ = −0.1 eV. The other parameters are the same as in case 1. We see in Figure 5 that the electron transfer rate drops strongly with the increase of the vacuum potential but the time scale is considerably slower than for Cases 1 − 2 due to the much larger vacuum-gap thickness. In this case, the final simulation time value is 0.1*μ*sec!

The above case studies reveal the influence of parameters such as distance between contacting materials, the Fermi levels, temperature, and effective masses. In particular, it is shown that the charge transfer rate is critically dependent on the distance (exponential decrease with increasing distance), a high Fermi level of the electron donor material and a low Fermi level of the electron acceptor material. Furthermore, a small effective mass increases the electron mobility and the tunneling transmission probability.

We point to that the general trends and influence of distance and Fermi levels that our model displays are captured by experimental results [45]. Recent theoretical models for contact electrification [15, 45] also compare well to the present more detailed modeling results.

It must be emphasized that the present model describes the first process of contact electrification and not the subsequent operation of a TENG mode. We refer the reader to recent work addressing TENG mode operation and power generation [46–48].

It is important to notice that our model demonstrates that the tunneling time may vary by several orders of magnitude (from picosecs to microsecs or millisecs) depending on model parameters. Therefore, for large separation of contacting materials, tunneling times can be of the same order of magnitude as typical triboelectric time constants.

## 9. Conclusions

A simple model is proposed for electron transfer between two solids. The model effectively demonstrates tunneling of electrons through the vacuum barrier and is applicable to both metals, semiconductors, and dielectrics. We use a simple effective-mass Hamiltonian for the electronic wavefunctions to calculate the electron transmission coefficient between two solids separated by a vacuum gap. The Tsu-Esaki equation is then used to compute the dynamics of loss-less electron tunneling and contact electrification. We demonstrate in three case studies that the strength of the tunneling process is very sensitive to the relative positions of the conduction-band edges of the two solids, the vacuum gap and potential, the Fermi levels and temperature of the solids, and the electron effective mass. Coulomb repulsion between electrons on the left and right material surfaces is accounted for in the tunneling dynamics.
